# Editorial

**Published:** 1874-02

**Authors:** 


### Editorial.

### *Are Lunatics Criminals?*

In the November number of the JOURNAL we took occasion, under the above caption, to call the attention of the County Commissioners to the urgent necessity of providing some suitable place or places for the safe keeping of lunatics pending the adjudication of their status, and previous to their transmission to an asylum by order of the County Court. Attention was directed to the great abuse, at present existing, of subjecting these unfortunate beings to incarceration as felons with felons, surrounded by the degrading and humiliating associations of the police station or the jail.

Another phase of the same subject has just been presented in the report of the Superintendent of the House of Correction and that of the City Physician included therein, published *in extenso* in the *Chicago Times* of January 15th; a thoughtful perusal of which again suggests the question, Are lunatics criminals? The phase of the subject comprehended by the question is a different one from that already presented. In that, we called attention to the outrage done to persons recognized as innocent, and yet treated as criminals, because they were so unfortunate as to be insane; in this, we would indicate that still more unfortunate class recognized as criminals and treated as such, who have helplessly drifted into crime, many of whom would even fulfill the old legal definition of insanity, for they know not right from wrong.

The opportunities for observation of this class, enjoyed by the two gentlemen to whose reports we have already referred, are ample to authorize them to speak *ex cathedra* upon the subject of the necessity which exists for distinguishing these helplessly criminal beings from those who are criminal maliciously. Their crimes are not so much voluntary acts as they are the necessary consequences of the reaction of their vicious surroundings upon organizations already degraded. The City Physician evidently comprehends the subject justly and thoroughly. Would it not be well to enlarge the scope of his duties to such an extent as to constitute him the municipal expert before the Magistrates' Courts, to aid those functionaries in discriminating correctly between willful criminals and helpless offenders, to the end that a classification might be made in their disposition, and the ends of public justice subserved, without involving a violation of the instincts of humanity or the teachings of philanthropy? The House of Correction might thus become a reformatory instead of a deformatory. H.

***The Board of Health.***

After having, on various occasions, animadverted upon the delinquencies of this soulless body, we did not expect to find ourselves, at this late day, arrayed among the ranks of its defenders. But in truth, while we can bear to see attacks made upon anything that has life, and the power to kick back, we can only regret the cruelty which abuses the dead. What has the Board of Health done to the new Mayor that he should vent his spite upon it so cruelly? Or, has he been refreshing his recollections of Æsop's fables, and proposes to play Jupiter and the Frogs? If so, give us king Stork, Oh, Jupiter! instead of king Log. H.

***The Premonitory Symptoms of Insanity,***

Is the title of an article published in the *Cincinnati Lancet and Observer*, of January, 1874, which bore so striking a resemblance to a paper published in this journal, of November, 1873, as to induce us to compare them. The only difference perceptible was a slight one between the name of the author appended to the *Lancet and Observer* article, and that subscribed to the original manuscript in our possession. It is true that there is no copyright in such matters; but still, gratitude, (not to speak of another still more necessary virtue,) demands a recognition of the sources of our benefits. H.

